# Acute Upper Gastrointestinal Bleeding is Associated With Poor Prognosis in Patients With Biliary Tract Cancer

**DOI:** 10.1002/cam4.72039

**Published:** 2026-06-14

**Authors:** Jan Christoph Schumacher, Sonja Lang, Philipp Kasper, Anna Martin, Dirk Waldschmidt, Martin Bürger, Ulrich Töx, Christoph Neumann‐Haefelin, Gabriel Allo

**Affiliations:** ^1^ Faculty of Medicine and University Hospital of Cologne, Department of Gastroenterology and Hepatology University of Cologne Cologne Germany

**Keywords:** biliary tract cancer, gastrointestinal hemorrhage, tumor‐associated bleeding, upper gastrointestinal bleeding

## Abstract

**Aim:**

Upper gastrointestinal bleeding (UGIB) is associated with an unfavorable outcome in cancer patients. In patients with biliary tract cancer (BTC), data on the incidence, management, and prognostic implications of UGIB are limited. This study investigated the prevalence, treatment efficacy, and prognostic factors associated with UGIB in BTC.

**Methods:**

This retrospective observational study included all patients with BTC and UGIB, treated at a tertiary care center between 2010 and 2023. Clinical, laboratory, endoscopic, and survival data were obtained from hospital records. Outcomes included short‐term rebleeding within 14 days and overall survival.

**Results:**

Among 1033 BTC patients, 37 (3.6%) experienced UGIB. The predominant etiologies were tumor‐associated bleeding due to tumor infiltration (27.0%), variceal bleeding (21.6%), and ulcer bleeding (16.2%). Endoscopic therapy was performed in 43.2% of cases, achieving hemostasis in 78.6% of actively bleeding patients. Short‐term rebleeding occurred in 28.6%. The 30‐day mortality following UGIB was 35.7%. Tumor‐associated UGIB correlated with significantly increased mortality (HR = 2.83, 95% CI 1.1–7.27, *p* = 0.030) and markedly reduced overall survival (28 vs. 90 days, *p* = 0.022).

**Conclusions:**

UGIB is a rare but severe complication in BTC. Tumor‐associated UGIB was associated with poor prognosis and markedly shortened survival, underscoring the necessity for careful clinical assessment and close follow‐up in this high‐risk cohort.

## Lay Summary

Upper gastrointestinal bleeding (UGIB) is a severe complication in patients with gastrointestinal cancer and is associated with poor outcomes. To date, no systematic investigation has examined the causes and clinical impact of UGIB in patients with biliary tract cancer. Our study shows that UGIB, although rare, most often occurs in advanced stages of the disease, is primarily caused by tumor infiltration or variceal bleeding, and is associated with markedly poor outcomes in this patient group. While endoscopic treatment can achieve initial hemostasis, rebleeding is frequent, emphasizing the need for close follow‐up and careful clinical management.

**Abbreviations**
APC: argon plasma coagulation
BTC: biliary tract cancer
CI: confidence interval
DAPT: dual antiplatelet therapy
FFP: fresh frozen plasma
HR: hazard ratio
ICU: intensive care unit
IMC: intermediate care unit
IQR: interquartile range
PRBC: packed red blood cell
UGIB: upper gastrointestinal bleeding

## 1 Introduction

Biliary tract carcinoma (BTC) is a rare malignancy, encompassing intrahepatic, perihilar, and distal cholangiocarcinoma as well as gallbladder carcinoma and ampullary carcinoma. Recent observations indicate a rising incidence of BTC in the Western countries, primarily driven by intrahepatic cholangiocarcinoma, which, with an incidence of 0.80–1.99 per 100,000 person‐years, now accounts for the second most common primary liver malignancy [1, 2, 3, 4]. Due to late diagnosis, curative surgical intervention is feasible in only about 22% of cases, and despite recent therapeutic advancements, the prognosis remains poor [3, 4, 5, 6].

While cholangitis and cachexia are the leading causes of death in patients with BTC, approximately 5% of patients die from acute gastrointestinal bleeding [7, 8]. Acute upper gastrointestinal bleeding (UGIB) is a medical emergency, with an incidence of 67–81 per 100,000 individuals per year [9, 10, 11]. Bleeding from malignancies in the gastrointestinal tract accounts for about 1%–5% of all UGIB cases, with a rising tendency, and typically occurs at advanced tumor stages [11, 12, 13].

In patients with BTC, UGIB may arise through heterogeneous mechanisms due to the diverse anatomical locations and growth patterns of these tumors. Ampullary carcinoma and distal cholangiocarcinoma may cause bleeding through direct tumor infiltration of the adjacent gastrointestinal tract. In contrast, perihilar or intrahepatic cholangiocarcinoma may predispose to bleeding through hemobilia or malignant vascular obstruction, which may contribute to portal hypertension and thus variceal hemorrhage, particularly in advanced stages of the disease [14, 15, 16].

Regardless of the bleeding source, gastrointestinal bleeding in cancer patients is associated with poor outcomes, with UGIB being linked to the worst prognosis [17, 18, 19, 20]. Although endoscopic therapy can be effective in achieving initial hemostasis in malignancy‐related UGIB, rebleeding remains common and mortality after tumor‐associated UGIB is high [11, 12, 13, 21]. Endoscopic management in BTC may be particularly challenging, as bleeding sources may not always be directly accessible, especially in the context of hemobilia or bleeding episodes due to portal hypertension, where different treatment strategies may be required.

Despite its clinical relevance, UGIB in patients with BTC remains poorly characterized. This study aimed to explore the prevalence and causes of UGIB in BTC, evaluate therapeutic management and clinical outcomes, and explore clinical factors associated with rebleeding and poor survival.

## 2 Methods

### 2.1 Patient Selection

This retrospective, single‐center observational study was conducted at the University Hospital of Cologne, Cologne, Germany, and included patients with histologically confirmed BTC who underwent esophagogastroduodenoscopy for clinically suspected UGIB between January 1, 2010, and December 31, 2023.

The study was approved by the Ethics Committee of the Faculty of Medicine, University of Cologne, Cologne, Germany (reference number: 25‐1285‐retro). The requirement for written informed consent was waived due to the retrospective, non‐interventional study design and the use of anonymized patient data.

UGIB was defined as clinically suspected bleeding, based on clinical signs of gastrointestinal bleeding such as hematemesis, melena, or hematochezia, together with endoscopic or radiologic evidence indicating an upper gastrointestinal bleeding source. Endoscopic evidence included active bleeding, stigmata of recent hemorrhage (clots, visible vessel, hematin), or an identifiable non‐actively bleeding lesion considered by the treating endoscopist to represent the likely bleeding source. In patients without active bleeding or evidence of recent hemorrhage on endoscopy, UGIB was considered confirmed if the clinical presentation was compatible with UGIB (such as hematemesis) and no other bleeding source was identified.

A bleeding event was defined as an episode of UGIB requiring diagnostic evaluation or hemostatic management. In patients with multiple admissions for UGIB during the study period, the initial presentation was defined as the index bleeding event.

The study population included all patients with histologically confirmed BTC who fulfilled the predefined criteria for UGIB during the study period. Exclusion criteria were: lack of histological confirmation of BTC, miscoded BTC or gastrointestinal bleeding diagnoses, UGIB outside the defined study period, insufficient documentation of the UGIB episode for further analysis, lower gastrointestinal bleeding without evidence of an upper gastrointestinal source, and iatrogenic or periprocedural bleeding events.

The institutional endoscopy database and hospital records were used to retrospectively evaluate eligible patients. Data on age, sex, BTC subtype (intra‐ or extrahepatic cholangiocarcinoma, gallbladder carcinoma, and ampullary carcinoma), age at first diagnosis, tumor stage at bleeding event, and previous antitumor therapy (prior surgery (radio‐) chemotherapy) were collected.

At the index bleeding event, bleeding stigmata (hematochezia, hematemesis, melena), vital signs, hemoglobin level, antiplatelet/anticoagulant medication, admission to the intermediate care (IMC) or intensive care unit (ICU), and transfusion of packed red blood cells (PRBCs) were assessed. Mortality data and cause of death were obtained from hospital records.

Bleeding etiology was assessed based on the documentation available in the institutional endoscopy database. Tumor‐associated UGIB was defined as an actively bleeding tumor or signs of recent hemorrhage during endoscopy without another identifiable bleeding source as the tumor.

Shock was defined as systolic blood pressure < 90 mmHg or heart rate exceeding systolic blood pressure. Rebleeding ≤ 14 days was classified as short‐term and > 14 days as long‐term. Successful hemostasis was defined as cessation of bleeding after endoscopic treatment.

### 2.2 Statistical Analysis

Analyses were performed using GraphPad Prism (version 10.2.2, GraphPad Software, Boston, Massachusetts, USA), R (version 4.50; R Core Team 2025, Vienna, Austria) within the RStudio environment (version 2025.05.1 + 513, RStudio Team (2025), PBC, Boston, MA), and Microsoft Excel (Microsoft Corporation, Redmond, WA, USA).

Overall survival was calculated from the time of the index UGIB to death from any cause. Patients without documented death were censored at the date of last recorded follow‐up. Patients undergoing curative tumor resection after the index bleeding event were excluded from subsequent overall survival analyses. Overall survival between patients with tumor‐associated and non‐tumor‐associated UGIB was compared using Kaplan–Meier analysis and the Mantel–Cox log‐rank test, with median survival reported with 95% confidence intervals.

The 30‐day mortality rate was calculated separately; patients without documented death and with last recorded follow‐up before day 30 were excluded from the 30‐day mortality analysis.

Univariable Cox regression analysis was performed to identify factors associated with short‐term rebleeding and overall survival after index UGIB. Patient selection criteria for the overall survival Cox regression analysis were identical to those used for the Kaplan–Meier analysis. For the analysis of short‐term rebleeding risk, patients were followed from index UGIB until rebleeding, death, last documented follow‐up, or day 14, whichever occurred first. Patients without rebleeding were censored at day 14 or earlier at death or last documented follow‐up. Patients undergoing operative tumor resection after index bleeding or patients for whom neither rebleeding status nor a valid censoring time could be determined were excluded from the analysis.

Variables that were statistically significant in the univariable analysis were further evaluated for potential confounding. Given the limited sample size and number of events, exploratory two‐variable Cox regression models were fitted for overall survival. Each adjusted model included the main variable identified in univariable analysis and one additional clinically relevant covariate at a time. These covariates included age at diagnosis, metastatic tumor stage, tumor recurrence, portal vein obstruction, transfusion of more than two PRBCs, and platelet count. The results were reported as hazard ratio (HR) with 95% confidence intervals (CI). A *p*‐value < 0.05 was considered statistically significant.

Patients' baseline characteristics were compared using the Wilcoxon rank‐sum test for continuous variables. Categorical variables were compared using Pearson's chi‐square test when expected cell counts exceeded six; otherwise, Fisher's exact test was used.

## 3 Results

**FIGURE 1:**
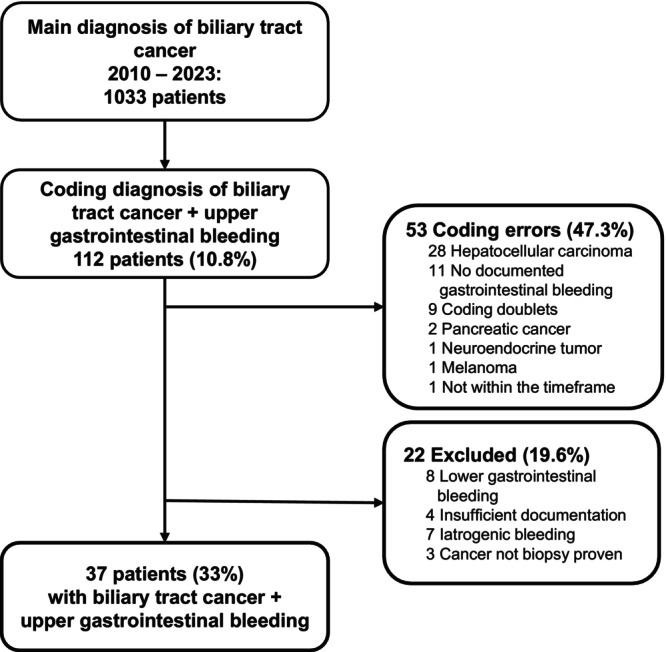
Study flow chart.

Of 1033 patients with BTC treated during the study period, 112 had a documented diagnosis of gastrointestinal bleeding (Figure 1). After the exclusion of 53 miscoded cases and 22 patients not meeting inclusion criteria, the final analysis included 37 patients with confirmed BTC and UGIB, representing an incidence of UGIB of 3.6% over a 13‐year observation period.

### 3.1 Bleeding Incidence and Patient Characteristics

The median age at tumor diagnosis was 68 years (interquartile range [IQR] 54–74 years), and 19 patients (51.4%) were female (Table 1). The most common tumor types were perihilar (40.5%) and intrahepatic cholangiocarcinoma (29.7%), followed by ampullary carcinoma (16.2%), distal cholangiocarcinoma (8.1%), and gallbladder carcinoma (5.4%).

**TABLE 1:** Characteristics of 37 patients with biliary tract cancer and upper gastrointestinal bleeding.

	*n* = 37 patients with BTC + UGIB, *n* (%)
Median age at diagnosis	68 years (IQR 54–74)
Median time between diagnosis and index bleeding event	259 days (IQR 111–858)
Gender—female/male	19/18 (51.4% / 48.6%)
Subtypes of biliary tract cancer	
Perihilar cholangiocarcinoma	15 (40.5%)
Intrahepatic cholangiocarcinoma	11 (29.7%)
Distal cholangiocarcinoma	3 (8.1%)
Ampullary carcinoma	6 (16.2%)
Gallbladder carcinoma	2 (5.4%)
Tumor stage at time of the bleeding event	
Resectable	4 (10.8%)
Locally advanced	12 (32.4%)
Metastatic	21 (56.8%)
Portal vein obstruction	
None	23 (62.2%)
Tumor‐associated	12 (32.4%)
Non‐tumor‐associated (thrombosis, stenosis)	2 (5.4%)
Liver cirrhosis	6 (16.2%)
Anticoagulant or dual antiplatelet therapy prior to the bleeding event	13 (35.1%)
Dual antiplatelet therapy	1 (2.7%)
Anticoagulant medication	12 (32.4%)
Previous antitumor therapy	
Operative resection	15 (40.5%)
Chemotherapy	23 (62.2%)
Radiotherapy	4 (10.8%)
Local ablative procedure	4 (10.8%)
No previous treatment	8 (21.6%)
Recurrent disease after resection	13 (35.1%)
Time from previous tumor‐directed therapy prior to UGIB, median (IQR), days	
Operative resection	653 days (IQR 368.5–1121.5)
Radiotherapy	177 days (IQR 175.2–559)
Local ablative procedure	366.5 days (IQR 16.8–937.8)
Time from last systemic therapy administration to UGIB, median (IQR), days	9.5 days (IQR 1–42.5)
Systemic antitumor therapy within 14 days before UGIB	14 (37.8%)
Tumor response to therapy at the time of the bleeding event	
No treatment	8 (21.6%)
Complete/partial remission	2 (5.4%)
Stable disease	2 (5.4%)
Progressive disease	17 (45.9%)
No staging under therapy	8 (21.6%)
Organ involvement at disease progression, *n*/*N* progressive disease (%)	Total, *n* = 17
Lymphatic	10 (58.8%)
Intrahepatic	14 (82.4%)
Gastrointestinal tract (duodenum/stomach)	3 (17.6%)
Other sites (pancreatic, renal, osseous, pulmonary, peritoneal)	7 (41.2%)
Not specified	1 (5.9%)

Abbreviations: BTC, biliary tract carcinoma; UGIB, upper gastrointestinal bleeding.

At the time of the index UGIB, most patients had an advanced tumor stage, with tumor metastases being present in over half of the patients (56.8%). The majority of patients had received prior antitumor therapy: 23 patients (62.2%) had received chemotherapy, and 15 patients (40.5%) had undergone surgical tumor resection, of whom 13 (35.1%) experienced recurrent disease.

The median interval between previous tumor‐directed therapy and index UGIB was 653 days (IQR 368.5–1121.5) for operative resection, 177 days (IQR 175.2–559) for radiotherapy, and 366.5 days (IQR 16.8–937.8) for local ablative procedures.

The median interval between the last administration of systemic therapy and index UGIB was 9.5 days (IQR 1–42.5). Before the onset of index bleeding, 14 patients (37.8%) had received chemotherapy within the preceding 2 weeks. Disease progression despite ongoing antitumor therapy was documented in 17 patients (45.9%) at restaging assessments. Among these, intrahepatic tumor progress was most common (82.4%). Eight patients (21.6%) had not yet received any antitumor therapy at the time of index UGIB.

The median interval between tumor diagnosis and UGIB was 259 days (IQR 111–858 days). At index UGIB, 12 patients (32.4%) were receiving anticoagulant medication, and one patient received dual antiplatelet therapy (2.7%).

### 3.2 UGIB Etiology and Endoscopic Findings

Melena (59.5%) was the most frequent clinical sign of bleeding upon presentation, followed by hematemesis (37.8%) (Table 2). At the time of the index bleeding, seven patients (18.9%) exhibited symptoms of shock. The median hemoglobin level was 73 g/L (IQR 62–80 g/L), and 62.2% of patients received PRBC transfusions, with seven patients (18.9%) receiving more than two units of PRBCs within the first 48 h. During the bleeding event, eight patients (21.6%) required admission to an intermediate (IMC) or intensive care unit (ICU) for hemodynamic monitoring.

**TABLE 2:** Clinical presentation and endoscopic findings.

	*n* = 37 patients with BTC + UGIB
Signs of bleeding, *n* (%)	
Melena	22 (59.5%)
Hematemesis	14 (37.8%)
Hematochezia	2 (5.4%)
Syncope	1 (2.7%)
Vital signs, median (IQR)	
Heart rate [bpm]	85 (IQR 76–96)
Systolic blood pressure [mmHg]	103 (IQR 99.2–116.5)
Symptoms of shock	7 (18.9%)
Laboratory parameters, median (IQR)	
Hemoglobin [g/L]	73 (IQR 62–80)
Platelet count [/μL]	195 (IQR 144–241)
Admission to the IMC/ICU, *n* (%)	8 (21.6%)
Transfusion of PRBC, *n* (%)	23 (62.2%)
Transfusion > 2 PRBCs	7 (18.9%)
Endoscopic bleeding stigmata, *n* (%)	
Active bleeding	14 (37.8%)
Signs of recent hemorrhage (hematin, blood clots, etc.)	15 (40.5%)
No bleeding stigmata	8 (21.6%)
Etiology of UGIB, *n* (%)	
Tumor bleeding	10 (27.0%)
Variceal hemorrhage	8 (21.6%)
Peptic ulcer	6 (16.2%)
Gastritis	4 (10.8%)
Hemobilia	1 (2.7%)
Unknown	5 (13.5%)
Others	3 (8.1%)
Endoscopic therapy performed, *n* (%)	Total, *n* = 16 (43.2%)
Clip placement	6/16 (37.5%)
Band ligature	5/16 (31.2%)
APC	3/16 (18.8%)
Injection therapy	1/16 (6.2%)
Hemostatic powder	1/16 (6.2%)
Other	1/16 (6.2%)
Conservative measurements	Total, *n* = 21 (56.8%)
Vitamin K supplementation	4/21 (19.0%)
Transfusion of FFP	2/21 (9.5%)
Radiological intervention after index bleeding	2 (5.4%)
Operative intervention after index bleeding	2 (5.4%)

Abbreviations: APC, argon plasma coagulation; BTC, Biliary tract carcinoma; FFP, fresh frozen plasma; IMC, intermediate care unit, ICU: intensive care unit; PRBC, packed red blood cell; UGIB, upper gastrointestinal bleeding.

The most frequent etiology of UGIB was tumor bleeding, caused by direct mucosal invasion (27.0%), followed by variceal hemorrhage (21.6%) and peptic ulcer bleeding (16.2%) (Table 2).

Active bleeding upon endoscopy was observed in 14 patients (37.8%). In 11 (78.6%) of these, endoscopic hemostasis was successful, whereas in the remaining three, the bleeding source was either inaccessible to or not suitable for endoscopic therapy as judged by the endoscopist (Table 3). One of these patients presented with hemobilia, one with hemorrhagic gastritis, and one exhibited a diffuse tumor infiltration of the duodenum.

**TABLE 3:** Therapeutic outcomes in relation to the bleeding status.

Outcome	Active bleeding with endoscopic therapy performed (*n* = 11)		Active bleeding without endoscopic therapy performed (*n* = 3)		Signs of recent hemorrhage (hematin, blood clots, etc.) (*n* = 15)		No bleeding stigmata (*n* = 8)		Total (*n* = 37)	
Endoscopic therapy performed	11	(100%)	0	(0%)	5	(33.3%)	0	(0%)	16	(43.2%)
Endoscopic hemostasis successful	11/11	(100%)	—		5/5	(100%)	—		16/16	(100%)
Conservative management	0	(0%)	3	(100%)	10	(66.7%)	8	(100%)	21	(56.8%)
Secondary operative intervention	0	(0%)	0	(0%)	1	(6.7%)	1	(12.5%)	2	(5.4%)
Secondary radiological intervention	0	(0%)	0	(0%)	2	(13.3%)	0	(0%)	2	(5.4%)
Symptoms of shock	2	(18.2%)	1	(33.3%)	3	(20%)	1	(12.5%)	7	(18.9%)
Transfusion at index bleeding, median (IQR)	2	(0.2–2)	2	(2, 3)	2	(0–2)	1	(0–2)	2	(0–2)
Transfusion > 2 PRBCs	2	(18.2%)	1	(33.3%)	3	(20%)	1	(12.5%)	7	(18.9%)
Short‐term rebleeding ≤ 14 days^a^	3/11	(27.3%)	0/3	(0%)	4/14	(28.6%)	3/7	(42.9%)	10/35	(28.6%)
Long‐term rebleeding > 14 days^a^	2/11	(18.2%)	0/3	(0%)	2/14	(14.3%)	0/7	(0%)	4/35	(11.4%)
30‐day mortality^a^, ^b^	3/7	(42.9%)	2/2	(100%)	5/13	(38.5%)	0/6	(0%)	10/28	(35.7%)
Median survival (days)^a^	90		22		34		Not reached		52	

Abbreviation: PRBC, packed red blood cell.

^a^ Patients who underwent curative tumor resection after index bleeding were excluded from subsequent survival and re‐bleeding analysis.

^b^ Patients with loss to follow‐up within 30 days were excluded from the 30‐day mortality analysis.

Stigmata of recent hemorrhage (e.g., hematin or intraluminal clots) were observed in another 15 patients (40.5%), with five receiving endoscopic treatment. Two had banding of esophageal varices, one received a metal clip for a Dieulafoy lesion, one underwent argon plasma coagulation (APC), and one had sclerosing therapy for gastric varices.

In eight patients (21.6%), endoscopy did not reveal active or recent bleeding despite reported clinical bleeding stigmata upon admission. A probable bleeding source was identified in four cases, while the remaining four remained unclear.

### 3.3 Hemostatic Management and Treatment Outcomes

If an endoscopic intervention was performed, the most frequently applied device was metal clips (37.5%), followed by band ligation for esophageal varices (31.2%) and APC (18.8%) (Table 2). One patient received a combination of clip placement and hemostatic powder, and another underwent sclerotherapy for fundal varices. The 21 patients (56.8%) who did not undergo endoscopic therapy received conservative treatment involving proton pump inhibitor therapy and coagulation optimization through vitamin K supplementation (19.0%) or transfusion of fresh frozen plasma (FFP) (9.5%).

The patient in whom hemobilia was identified as the bleeding source was managed conservatively and did not undergo endoscopic retrograde cholangiopancreatography.

Two patients required radiological intervention (Table 2). One underwent arterial embolization for hemorrhage, and one had early transjugular intrahepatic portosystemic shunt placement for variceal bleeding.

Furthermore, two patients underwent surgical tumor resection following index UGIB. These patients were excluded from survival analysis.

Patients with active bleeding had the highest demand for PRBC transfusions, with three (21.4%) patients receiving more than two PRBCs within 48 h (Table 3). Overall, seven patients received more than two PRBCs in the first 48 h, of whom three (42.9%) showed symptoms of shock.

Short‐term rebleeding (≤ 14 days) occurred in 10 of 35 patients (28.6%). The highest short‐term rebleeding rate was observed in patients without stigmata of recent hemorrhage (42.9%). Long‐term rebleeding was most frequent among patients with active bleeding treated endoscopically (18.2%).

The highest 30‐day mortality rate was recorded in patients with active bleeding in whom endoscopic therapy was not possible (100%), corresponding to the lowest median survival of 22 days. Conversely, patients with active bleeding who underwent successful endoscopic therapy had the most favorable outcomes, with a median survival of 90 days.

### 3.4 Tumor‐Associated Bleeding

Ten patients experienced UGIB due to tumor infiltration of the gastrointestinal wall, with three (30%) experiencing recurrent bleeding events, resulting in a total of 15 episodes of tumor‐associated UGIB. These patients suffered from perihilar (5/10), ampullary (3/10), or intrahepatic cholangiocarcinoma (2/10).

Although these differences did not reach statistical significance, patients with tumor‐associated UGIB were older (median 70.5 vs. 65 years) and more frequently had metastatic disease compared to those with non–tumor‐associated UGIB (Tables S1 and S2). Specifically, eight (80%) patients had metastatic disease and two (20%) had locally advanced disease, compared with 13 (48.1%) and 10 (37%) patients in the non‐tumor‐associated group, respectively. Gastrointestinal tract infiltration was documented in three patients with metastatic disease and tumor‐associated UGIB, whereas no gastrointestinal tract infiltration was observed among patients with metastatic disease and non‐tumor‐associated UGIB. Systemic antitumor therapy within 2 weeks prior to the bleeding event was more common in the tumor‐associated UGIB group (50% vs. 33.3%), and the median interval from last administration of systemic therapy was shorter (3 days (IQR 0–7.5) vs. 20.5 days (IQR 1.8–242)).

Among all the parameters assessed, only platelet count differed significantly between the two groups and was significantly lower in patients with tumor‐associated UGIB (136.5/μl vs. 224/μl, *p* = 0.02).

Endoscopic therapy was performed in three patients (30%) with tumor‐associated bleeding and in 13 patients (48.1%) with non‐tumor‐associated UGIB, whereas the remaining patients were managed conservatively.

Treatment strategies for recurrent episodes of tumor‐associated UGIB included conservative management (53.3%), endoscopic therapy (40.0%), and radiologic intervention with radioembolization (6.7%). Endoscopic hemostasis was most commonly achieved using hemostatic powder (13.3%), clip application (13.3%) or a combination of both (6.7%) (Figure S1). Median time to rebleeding was 13 days (IQR 5.8–42.3 days).

### 3.5 Short‐Term Bleeding Recurrence

After index UGIB, 10 patients experienced short‐term rebleeding within 14 days. In univariable Cox regression analysis, none of the tested variables was significantly associated with short‐term rebleeding. Admission to the IMC/ICU (HR 2.73; 95% CI 0.73–10.19, *p* = 0.135) and tumor recurrence (HR 2.37; 95% CI 0.63–8.89, *p* = 0.200) showed the highest hazard ratios, but did not reach statistical significance (Table 4).

**TABLE 4:** Univariable Cox regression analysis of risk factors associated with bleeding recurrence within the next 14 days.

	Hazard ratio	95% CI	*p*
Age at diagnosis	1.01	0.96–1.07	0.665
Gender	0.91	0.24–3.37	0.882
Metastatic tumor stage	0.74	0.2–2.77	0.656
Tumor recurrence	2.37	0.63–8.89	0.200
Tumor‐associated UGIB	0.73	0.15–3.52	0.696
Portal vein obstruction	0.91	0.23–3.63	0.889
Systemic therapy within 14 days before UGIB	0.65	0.16–2.61	0.544
Anticoagulant therapy/DAPT	0.47	0.1–2.27	0.349
Symptoms of shock	1.99	0.44–9.01	0.373
Admission to the IMC/ICU	2.73	0.73–10.19	0.135
Transfusion	2.07	0.43–9.95	0.366
Transfusion > 2 PRBCs	1.13	0.23–5.47	0.877
Conservative therapy	0.5	0.14–1.88	0.308
Endoscopic hemostasis	1.98	0.53–7.4	0.308

Abbreviations: DAPT, dual antiplatelet therapy; ICU, intensive care unit; IMC, intermediate care unit; PRBC, packed red blood cell; UGIB, upper gastrointestinal bleeding.

### 3.6 Survival

UGIB accounted for 9% of deaths in the study, being the primary cause of death in two cases out of 22 with a documented cause of death.

Patients without curative tumor resection following UGIB had a 30‐day mortality of 35.7% and a median overall survival of 52 days (Table 3 and Figure S2). In univariable Cox regression analysis, Tumor‐associated UGIB was associated with a worse overall survival (HR 2.83, 95% CI 1.10–7.27; *p* = 0.030) (Table 5). Metastatic tumor stage showed an increased hazard ratio of death as well, but did not reach statistical significance (HR 2.71, 95% CI 0.99–7.40; *p* = 0.052).

**TABLE 5:** Univariable Cox regression analysis of risk factors associated with overall survival.

	Hazard ratio	95% CI	*p*
Age at diagnosis	0.96	0.92–1	0.053
Gender	1.54	0.62–3.84	0.354
Metastatic tumor stage	2.71	0.99–7.4	0.052
Tumor recurrence	1.3	0.53–3.21	0.563
Portal vein obstruction	1.64	0.67–3.99	0.276
Tumor‐associated UGIB	**2.83**	**1.1–7.27**	**0.030**
Bleeding recurrence	0.78	0.28–2.16	0.638
Chemotherapy within the last 2 weeks	0.84	0.35–2.05	0.706
Anticoagulant therapy/DAPT	1.02	0.4–2.61	0.962
Symptoms of shock	1.93	0.61–6.08	0.260
Admission to IMC/ICU	1.57	0.62–4.01	0.345
Transfusion	0.7	0.28–1.77	0.457
Transfusion > 2 PRBCs	0.3	0.07–1.33	0.114
Conservative therapy	1.25	0.5–3.1	0.629
Endoscopic hemostasis	0.8	0.32–1.98	0.629

*Note:* Bold font indicates statistical significance at *p* < 0.05.

Abbreviations: DAPT, dual antiplatelet therapy; ICU, intensive care unit; IMC, intermediate care unit; PRBC, packed red blood cell; UGIB, upper gastrointestinal bleeding.

To assess the robustness of the association between tumor‐associated UGIB and overall survival, exploratory adjusted Cox regression models were performed with one additional clinically relevant covariate at a time. Tumor‐associated UGIB remained associated with increased mortality in models adjusted for age at diagnosis, portal vein obstruction, and tumor recurrence. When adjusted for metastatic tumor stage or transfusion of more than two PRBCs, the hazard ratios remained elevated but did not reach statistical significance (Table S3). These findings were interpreted as exploratory given the limited sample size and broad confidence intervals.

**FIGURE 2:**
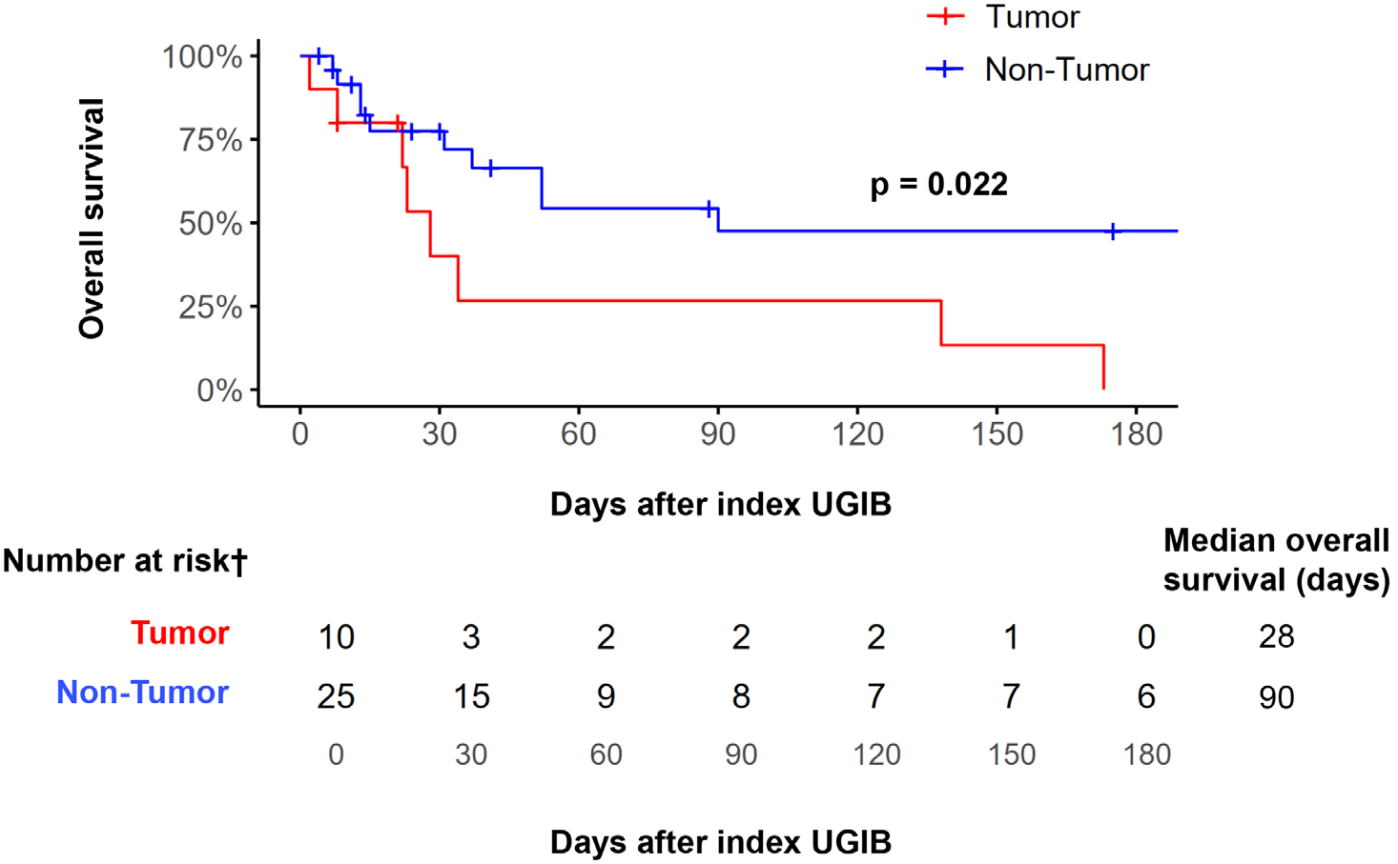
Overall survival is significantly lower in patients with tumor‐associated upper gastrointestinal bleeding. Cumulative curves of overall survival in patients with tumor‐associated UGIB and non‐tumor‐associated UGIB. The *x*‐axis was truncated at 180 days. ^†^Patients who underwent curative tumor resection after index bleeding were excluded from subsequent survival and re‐bleeding analysis. UGIB, upper gastrointestinal bleeding.

In direct comparison, patients with tumor‐associated UGIB exhibited significantly reduced survival rates compared to those with non‐tumor‐related UGIB (median overall survival 28 days vs. 90 days, log‐rank test *p* = 0.022) (Figure 2).

## 4 Discussion

In this study, UGIB occurred in 3.6% of patients with BTC over a 13‐year period and was associated with poor clinical outcomes. Tumor bleeding and variceal hemorrhage were the most common causes of UGIB. Although endoscopic therapy achieved initial hemostasis in selected patients, rebleeding occurred in approximately one‐third of patients. The overall prognosis following index UGIB was poor, with a median overall survival of 52 days. Mortality rates were especially high in patients with tumor‐associated UGIB (median overall survival = 28 days vs. 90 days).

To date, the incidence and etiology of gastrointestinal bleeding in BTC have not yet been extensively analyzed. The observed overall bleeding rate of 3.6% in our cohort is comparable to a previous study analyzing the US National Inpatient Database [22]. Here, the authors observed a gastrointestinal bleeding rate of 5%–6% without differentiating between upper and lower gastrointestinal bleeding or tumor‐associated UGIB. Another study by Kimura‐Seto et al. identified gastrointestinal bleeding as the cause of death in approximately 5% of patients with BTC, while in our study UGIB accounted for 9% of deaths [8]. This higher rate is likely due to the selective inclusion of patients with gastrointestinal bleeding in our study population.

Although UGIB is a relatively infrequent event in BTC, its clinical relevance is underscored by the high mortality rates observed. In our cohort, UGIB was associated with a poor prognosis regardless of the bleeding cause (overall 30‐day mortality rate of 35.7%). This may be linked to an advanced disease stage, as approximately half of the patients had metastatic disease and demonstrated progression under previous oncologic therapy at the time of index bleeding.

Patients with tumor‐associated UGIB experienced markedly poorer outcomes, with a significantly shorter median survival (28 days) compared with those with non‐tumor‐associated UGIB (90 days). Although not statistically significant, patients with tumor‐associated UGIB tended to be older, had more advanced disease, and were more heavily pretreated, which may have further contributed to the poor prognosis in this subgroup. Prior surgery and radiotherapy were generally remote from UGIB. Local ablative therapy occurred shortly before UGIB in one patient, but the number of cases was too small for meaningful inference. We therefore found no clear temporal association between prior surgery, radiotherapy, or local ablative therapy and UGIB, whereas recent systemic therapy remained the treatment modality most closely preceding UGIB, especially in tumor‐associated cases.

The last administered systemic therapy regimen before UGIB was heterogeneous in both groups, and no clear pattern suggesting regimen‐specific bleeding risk was observed. Capecitabine‐based therapy was the most frequent last regimen in both patients with tumor‐associated UGIB (30%) and those with non–tumor‐associated UGIB (22.2%). One patient with tumor‐associated UGIB had received gemcitabine + cisplatin in combination with durvalumab before the bleeding event.

In univariable Cox regression, tumor‐associated UGIB was significantly associated with worse overall survival (HR 2.83, 95% CI 1.10–7.27; *p* = 0.030). This association was also observed in several exploratory adjusted two‐variable Cox regression models (Table S3). However, tumor‐associated UGIB did not retain statistical significance after adjustment for metastatic tumor stage (HR 2.33, 95% CI 0.88–6.13; *p* = 0.087), transfusion requirement of more than two PRBCs (HR 2.45, 95% CI 0.95–6.33; *p* = 0.064) or platelet count (HR 2.66, 95% CI 0.86–8.27; *p* = 0.090). Metastatic tumor stage reflects overall disease burden and is strongly associated with poor prognosis, whereas transfusion requirement may represent a marker of bleeding severity. The lower platelet counts observed in patients with tumor‐associated UGIB may reflect treatment‐related myelosuppression, given the shorter interval between last systemic therapy and index UGIB in this group (3 days vs. 20.5 days) and therefore may also reflect disease burden as well as an additional factor of vulnerability to bleeding. Thus, the observed association between tumor‐associated UGIB and overall survival may partly reflect advanced tumor burden, bleeding severity and hematologic vulnerability. However, given the small sample size, these results should be interpreted cautiously.

Nevertheless, these findings support the clinical relevance of tumor‐associated bleeding in BTC. Tumor‐associated UGIB is associated with advanced disease and may represent a hallmark of severe clinical deterioration, as treatment of these bleedings is clinically challenging due to the limited effect of conservative and interventional treatment measures in this subgroup. This is consistent with previous studies analyzing other gastrointestinal malignancies, where tumor bleeding was also associated with dismal outcomes [12, 13, 18, 21].

To our knowledge, no detailed characterization of the etiology of UGIB in patients with BTC has been published to date. While a limited number of case reports have identified hemobilia as a potential cause of UGIB in BTC patients [23, 24], and others have reported UGIB in the setting of antitumor therapy such as surgical resection or radiotherapy [25, 26], comprehensive data on this topic are currently lacking. In the present cohort, tumor bleeding and variceal hemorrhage were the predominant causes of UGIB, while spontaneous hemobilia was infrequently observed (2.7%). The high rates of tumor‐associated UGIB were comparable to other studies on bleeding in different gastrointestinal cancer subtypes [13, 18, 21]. The occurrence of variceal hemorrhage has been predominantly described in patients with hepatocellular carcinoma [27, 28, 29], and has only been recently described as a rare source of bleeding in BTC patients by Ma et al., who were also able to show that the presence of esophagogastric varices was associated with a poorer prognosis in patients with BTC [16]. Of the eight patients with variceal bleeding in our study, only 37.5% had a confirmed diagnosis of liver cirrhosis, while 37.5% of patients developed variceal hemorrhage in the context of non‐cirrhotic portal hypertension due to malignant portal vein infiltration. These findings are consistent with the results of a previous study by Lim et al. which identified tumorous portal vein infiltration as a risk factor for variceal bleeding in hepatocellular carcinoma [28].

Endoscopic therapy proved effective in achieving initial hemostasis in selected patients in whom endoscopic intervention was considered technically feasible. Endoscopic clip placement and band ligation were the most frequently used procedures, whereas hemostatic powder was the predominant therapy for tumor‐associated UGIB. However, approximately half of the patients received conservative medical treatment only. This likely reflects the high proportion of inactive bleeding at the time of index endoscopy, as less than half of patients (37.8%) presented with active bleeding, of whom one‐fifth were deemed unsuitable for endoscopic intervention.

Despite the high success rate of endoscopic therapy in achieving initial hemostasis, rebleeding remained frequent, occurring in approximately one‐third of cases, indicating the persistent vulnerability of this patient population and the limited durability of endoscopic bleeding control in this high‐risk cohort. However, no reliable predictor of short‐term rebleeding was identified in univariable Cox regression analysis. The need for an admission to an intermediate care or intensive care unit as well as tumor recurrence after operative resection showed an association with early rebleeding but did not reach statistical significance.

Our data do not demonstrate superiority of endoscopic therapy over conservative management with regard to rebleeding or mortality. However, this comparison is limited by the retrospective design, as treatment allocation depended on bleeding activity, lesion characteristics, technical feasibility, and the treating endoscopist's assessment, resulting in substantial confounding by indication.

Therefore, these findings should not be interpreted as evidence against endoscopic therapy, as previous data suggest a mortality benefit in patients with primary hepatic and biliary tract cancer and gastrointestinal bleeding [22], but rather as an indication that durable bleeding control remains challenging in this high‐risk cohort despite successful immediate hemostasis in selected patients.

Several limitations of our study must be acknowledged. Despite screening a large cohort of BTC patients, the final number of patients with confirmed UGIB was small, reflecting the rarity of this clinical event. This limited statistical power and precluded robust multivariable adjustment, competing‐risk models, and formal analysis of subgroup‐specific or treatment‐background‐specific effects. As a result, residual confounding cannot be fully excluded. Patients undergoing curative tumor resection after the index bleeding were excluded from overall survival analysis, as curative tumor resection may have altered the subsequent disease course and prognosis. This exclusion, however, may have also influenced survival estimates, although the quantitative impact is likely limited given the small number of affected patients. Consequently, regression analyses should be regarded as exploratory, and adjusted Cox regression analyses were constrained to two‐variable models to mitigate the risk of overfitting.

Furthermore, the single‐center and retrospective nature of our study limits the generalizability of these findings. This study was conducted at a tertiary care center with significant expertise in the medical treatment and endoscopic complication management of patients with BTC. Therefore, our findings may not fully translate to other healthcare settings. External validation in larger multicenter cohorts will be important to confirm the reproducibility of the prognostic associations observed in this study.

## 5 Conclusions

To our knowledge, this is the first systematic investigation of the etiology, management, and outcomes of UGIB in a large cohort of patients with BTC. UGIB is a rare but clinically relevant complication that predominantly occurs in advanced disease stages and is associated with high rebleeding rates and poor survival. While endoscopic therapy achieved initial hemostasis in selected patients, recurrent bleeding remained frequent, indicating limited durability of bleeding control in this high‐risk population. Tumor‐associated UGIB was associated with particularly poor prognosis and may mark a clinically vulnerable subgroup of patients with advanced disease, for whom careful clinical assessment and close follow‐up after the initial bleeding episode appear warranted.

## Author Contributions

**Jan Christoph Schumacher:** conceptualization, methodology, formal analysis, investigation, writing – original draft, writing – review and editing. **Sonja Lang:** conceptualization, methodology, formal analysis, writing – review and editing. **Philipp Kasper:** writing – review and editing. **Anna Martin:** writing – review and editing. **Dirk Waldschmidt:** writing – review and editing. **Marting Bürger:** writing – review and editing. **Ulrich Töx:** writing – review and editing. **Christoph Neumann‐Haefelin:** conceptualization, writing – review and editing, supervision. **Gabriel Allo:** conceptualization, methodology, investigation, writing – original draft, writing – review and editing, supervision, formal analysis.

## Funding

This research received no external funding.

## Ethics Statement

This study was approved by the Ethics Committee of the Faculty of Medicine, University of Cologne, Cologne, Germany (reference number: 25‐1285‐retro). The requirement for written informed consent was waived by the ethics committee due to the retrospective, non‐interventional study design and the use of anonymized patient data.

## Conflicts of Interest

The authors declare no conflicts of interest.

## Supporting information


**Table S1:** Comparison of baseline characteristics, bleeding presentation and initial management of patients with tumor‐associated UGIB and non‐tumor‐associated UGIB.
**Table S2:** Comparison of tumor burden and treatment background of patients with tumor‐associated UGIB and non‐tumor‐associated UGIB.
**Table S3:** Exploratory adjusted two‐variable Cox regression models for overall survival with tumor‐associated UGIB as the main exposure.

**Figure S1:** Treatment strategies in tumor‐associated UGIB.
**Figure S2:** Overall survival following UGIB in patients with BTC.

## Data Availability

The data that support the findings of this study are available on request from the corresponding author. The data are not publicly available due to privacy or ethical restrictions.
