# Supplementary material for: Acute Upper Gastrointestinal Bleeding is Associated With Poor Prognosis in Patients With Biliary Tract Cancer
Source: Cancer Med. 2026 Jun 14;15(6):e72039. doi: 10.1002/cam4.72039 (PMC13265610; doi:10.1002/cam4.72039)
Supplement: Supplementary file 1 — Table S1: Comparison of baseline characteristics, bleeding presentation and initial management of patients with tumor‐associated UGIB and non‐tumor‐associated UGIB. Table S2: Comparison of tumor burden and treatment background of patients with tumor‐associated UGIB and non‐tumor‐associated UGIB. Table S3: Exploratory adjusted two‐variable Cox regression models for overall survival with tumor‐associated UGIB as the main exposure. [file CAM4-15-e72039-s001.doc]

**Suppl. Table 1. Comparison of baseline characteristics, bleeding presentation and initial management of patients with tumor-associated UGIB and non-tumor-associated UGIB**

|  | **Tumor-associated UGIB† N = 10** | **Non-tumor-associated UGIB† N = 27** | **p-value** |
| --- | --- | --- | --- |
| Median age at diagnosis | 70.5 years (IQR 60.5–73.5) | 65 years (IQR 53.5–74.5) | 0.7 |
| Median time between diagnosis and index bleeding event | 254 days (IQR 140–286) | 390 days (IQR 99.5–974) | 0.4 |
| Gender - female / male | 5/5 (50%/50%) | 14/13 (51.9%/48.1%) | >0.9 |
| Subtypes of biliary tract cancer |  |  | 0.5 |
| Perihilar cholangiocarcinoma | 5 (50%) | 10 (37%) |  |
| Intrahepatic cholangiocarcinoma | 2 (20%) | 9 (33.3%) |  |
| Distal cholangiocarcinoma | 0 (0%) | 3 (11.1%) |  |
| Ampullary carcinoma | 3 (30%) | 3 (11.1%) |  |
| Gallbladder carcinoma | 0 (0%) | 2 (7.4%) |  |
| Signs of bleeding, n (%) |  |  |  |
| Melena | 6 (60%) | 16 (59.3%) | >0.9 |
| Hematemesis | 3 (30%) | 11 (40.7%) | 0.7 |
| Hematochezia | 1 (10%) | 1 (3.7%) | 0.4 |
| Syncope | 0 (0%) | 1 (3.7%) | >0.9 |
| Vital signs, median (IQR) |  |  |  |
| Heart rate [bpm] | 88 (IQR 82.5–97.5) | 80 (IQR 70.8–96) | 0.3 |
| Systolic blood pressure [mmHg] | 101 (IQR 100–115) | 104 (IQR 90–120) | >0.9 |
| Symptoms of shock | 1 (10%) | 6 (22.2%) | 0.6 |
| Laboratory parameters, median (IQR) |  |  |  |
| Hemoglobin [g/L] | 74 (IQR 64–79) | 73 (IQR 63–82) | >0.9 |
| Platelet count [/µL] | 136.5 (IQR 90.2–178.2) | 224 (IQR 167–255.5) | 0.02 |
| Anticoagulant or dual antiplatelet therapy prior to the bleeding event | 5 (50%) | 8 (29.6%) | 0.3 |
| Liver cirrhosis | 1 (10%) | 5 (18.5%) | >0.9 |
| Admission to the IMC‡ / ICU§, n (%) | 2 (20%) | 6 (22.2%) | >0.9 |
| Transfusion of PRBC, n (%) | 7 (70%) | 16 (59.3%) | 0.4 |
| Transfusion >2 PRBCs¶ | 1 (10%) | 6 (22.2%) | 0.7 |
| Conservative therapy only | 7 (70%) | 14 (51.9%) | 0.5 |
| Endoscopic therapy | 3 (30%) | 13 (48.1%) | 0.5 |

Abbreviations: †UGIB: upper gastrointestinal bleeding, ‡ IMC: intermediate care unit, § ICU: intensive care unit, ¶ PRBC: packed red blood cell.

**Suppl. Table 2. Comparison of tumor burden and treatment background of patients with tumor-associated UGIB and non-tumor-associated UGIB.**

|  | **Tumor-associated UGIB† N = 10** | **Non-tumor-associated UGIB† N = 27** | **p-value** |
| --- | --- | --- | --- |
| Tumor stage at index UGIB† |  |  | 0.2 |
| Resectable | 0 (0%) | 4 (14.8%) |  |
| Locally advanced | 2 (20%) | 10 (37%) |  |
| Metastatic | 8 (80%) | 13 (48.1%) |  |
| Sites of metastatic disease at index UGIB†, n/N metastatic (%) | Metastatic, n = 8 | Metastatic, n = 13 |  |
| Lymphatic | 4 (50%) | 9 (69.2%) |  |
| Intrahepatic | 5 (62.5%) | 11 (84.6%) |  |
| Gastrointestinal tract (duodenum/stomach) | 3 (37.5%) | 0 (0%) |  |
| Other sites (pancreatic, renal, osseous, pulmonary, peritoneal) | 4 (50%) | 7 (53.8%) |  |
| Portal vein obstruction |  |  | >0.9 |
| None | 7 (70%) | 16 (59.3%) |  |
| Tumor-associated | 3 (30%) | 9 (33.3%) |  |
| Non-tumor-associated (thrombosis, stenosis) | 0 (0%) | 2 (7.4%) |  |
| Tumor response to therapy at the time of the bleeding event |  |  | >0.9 |
| No treatment | 2 (20%) | 6 (22.2%) |  |
| Complete / partial remission | 0 (0%) | 2 (7.4%) |  |
| Stable disease | 0 (0%) | 2 (7.4%) |  |
| Progressive disease | 5 (50%) | 12 (44.4%) |  |
| No staging under therapy | 3 (30%) | 5 (18.5%) |  |
| Previous tumor-directed therapy prior to UGIB† |  |  |  |
| Operative resection | 3 (30%) | 12 (44.4%) | 0.5 |
| Recurrent disease after resection | 3 (30%) | 10 (37%) | >0.9 |
| Radiotherapy | 1 (10%) | 3 (11.1%) | >0.9 |
| Local ablative procedure | 1 (10%) | 3 (11.1%) | >0.9 |
| Any prior systemic therapy | 6 (60%) | 17 (63%) | >0.9 |
| No previous tumor-directed treatment | 2 (20%) | 6 (22.2%) | >0.9 |
| Time from previous tumor-directed therapy prior to UGIB†, median (IQR), days |  |  |  |
| Operative resection | 653 days (IQR 451–749) | 696 days (IQR 415.8–1215.2) | 0.8 |
| Radiotherapy | 177 days (IQR 177–177) | 177 days (IQR 173.5–941) | >0.9 |
| Local ablative procedure | 17 days (IQR 17–17) | 366 days (IQR 191–541) | >0.9 |
| Time from last systemic therapy administration to UGIB†, median (IQR), days | 3 days (IQR 0–7.5) | 20.5 days (IQR 1.8–242) | 0.08 |
| Systemic therapy within 14 days before UGIB† | 5 (50%) | 9 (33.3%) | 0.4 |
| Time from initiation of last systemic therapy regimen to UGIB†, median (IQR), days | 42 days (IQR 22–163.2) | 81 days (IQR 60.5–171.8) | 0.2 |
| Last systemic therapy regimen before UGIB† |  |  | 0.4 |
| Gemcitabine+ cisplatin + durvalumab | 1 (10%) | 0 (0%) |  |
| Gemcitabine + cisplatin-based therapy | 0 (0%) | 4 (14.8%) |  |
| Gemcitabine + taxane-based therapy | 0 (0%) | 2 (7.4%) |  |
| Gemcitabine ± tyrosine kinase inhibitor | 2 (20%) | 3 (11.1%) |  |
| Capecitabine ± tyrosine kinase inhibitor | 3 (30%) | 6 (22.2%) |  |
| Other | 0 (0%) | 2 (7.4%) |  |
| Therapy line before UGIB†, median (IQR) | 2 (IQR 1.2–2.8) | 2 (IQR 1–3) | >0.9 |

Abbreviations: †UGIB: upper gastrointestinal bleeding.

**Suppl. Table 3. Exploratory adjusted two-variable Cox regression models for overall survival with tumor-associated UGIB as the main exposure.**

| **Variable** | **Hazard Ratio** | **95% CI** | **p-value** |
| --- | --- | --- | --- |
| **Unadjusted** | **2.83** | **1.1 - 7.27** | **0.030** |
| **Adjusted for age** | **3.35** | **1.27 - 8.85** | **0.015** |
| Adjusted for metastatic tumor stage | 2.33 | 0.88 - 6.13 | 0.087 |
| **Adjusted for tumor recurrence** | **3.14** | **1.18 - 8.35** | **0.022** |
| **Adjusted for portal vein obstruction** | **3.8** | **1.36 - 10.63** | **0.011** |
| Adjusted for transfusion >2 PRBCs† | 2.45 | 0.95 - 6.33 | 0.064 |
| Adjusted for platelet count | 2.66 | 0.86 - 8.27 | 0.090 |

Note: Bold font indicates statistical significance at p < 0.05.

Abbreviations: † PRBC: packed red blood cell
