# Supplementary material for: Acute Upper Gastrointestinal Bleeding is Associated With Poor Prognosis in Patients With Biliary Tract Cancer
Source: Cancer Med. 2026 Jun 14;15(6):e72039. doi: 10.1002/cam4.72039 (PMC13265610; doi:10.1002/cam4.72039)
Supplement: Supplementary file 2 — Figure S1: Treatment strategies in tumor‐associated UGIB. Figure S2: Overall survival following UGIB in patients with BTC. [file CAM4-15-e72039-s002.pptx]

## Slide 1
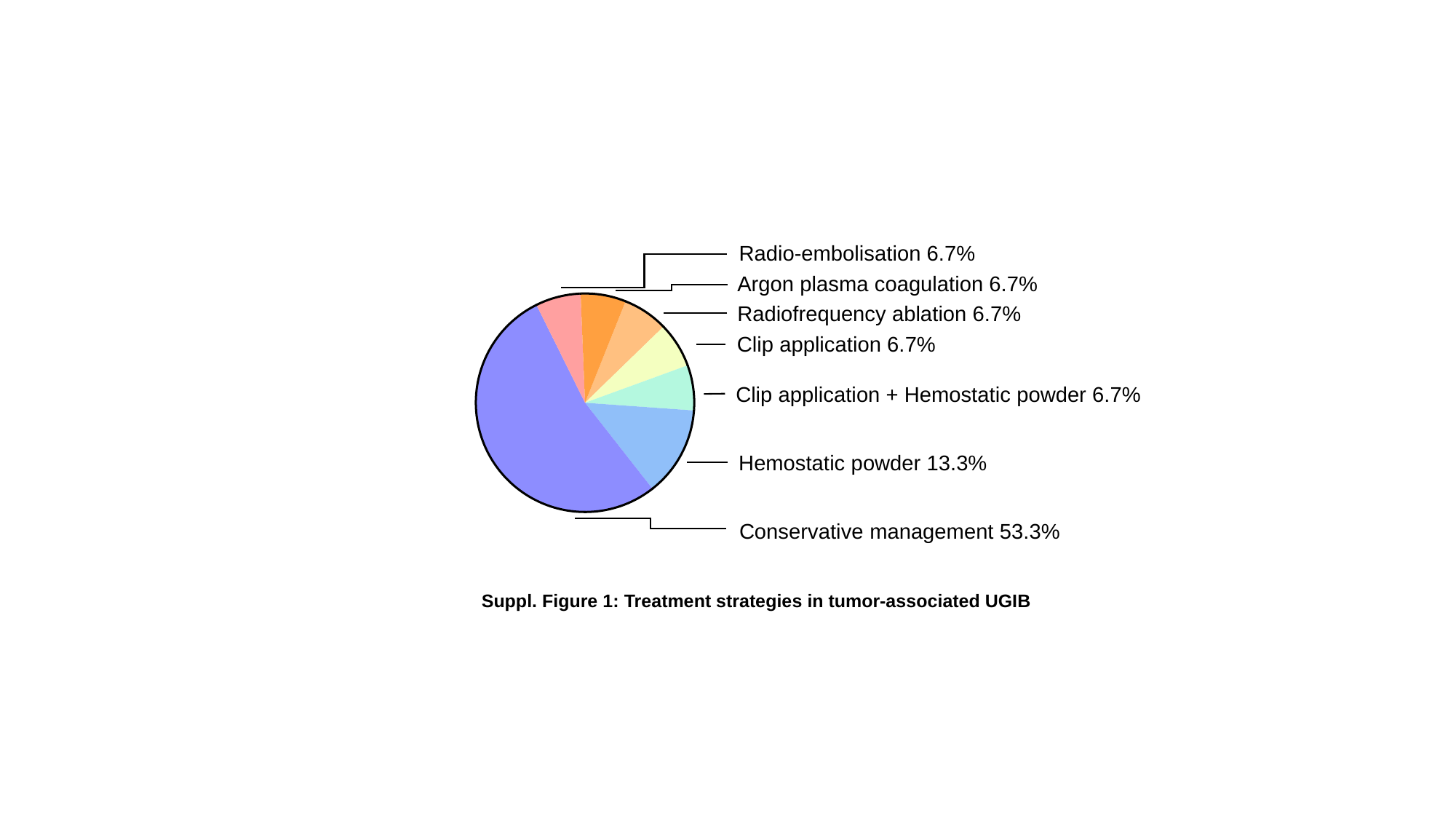

Radio-embolisation 6.7%
Argon plasma coagulation 6.7%
Clip application 6.7%
Hemostatic powder 13.3%
Conservative management 53.3%
Radiofrequency ablation 6.7%
Clip application + Hemostatic powder 6.7%
Suppl. Figure 1: Treatment strategies in tumor-associated UGIB

## Slide 2
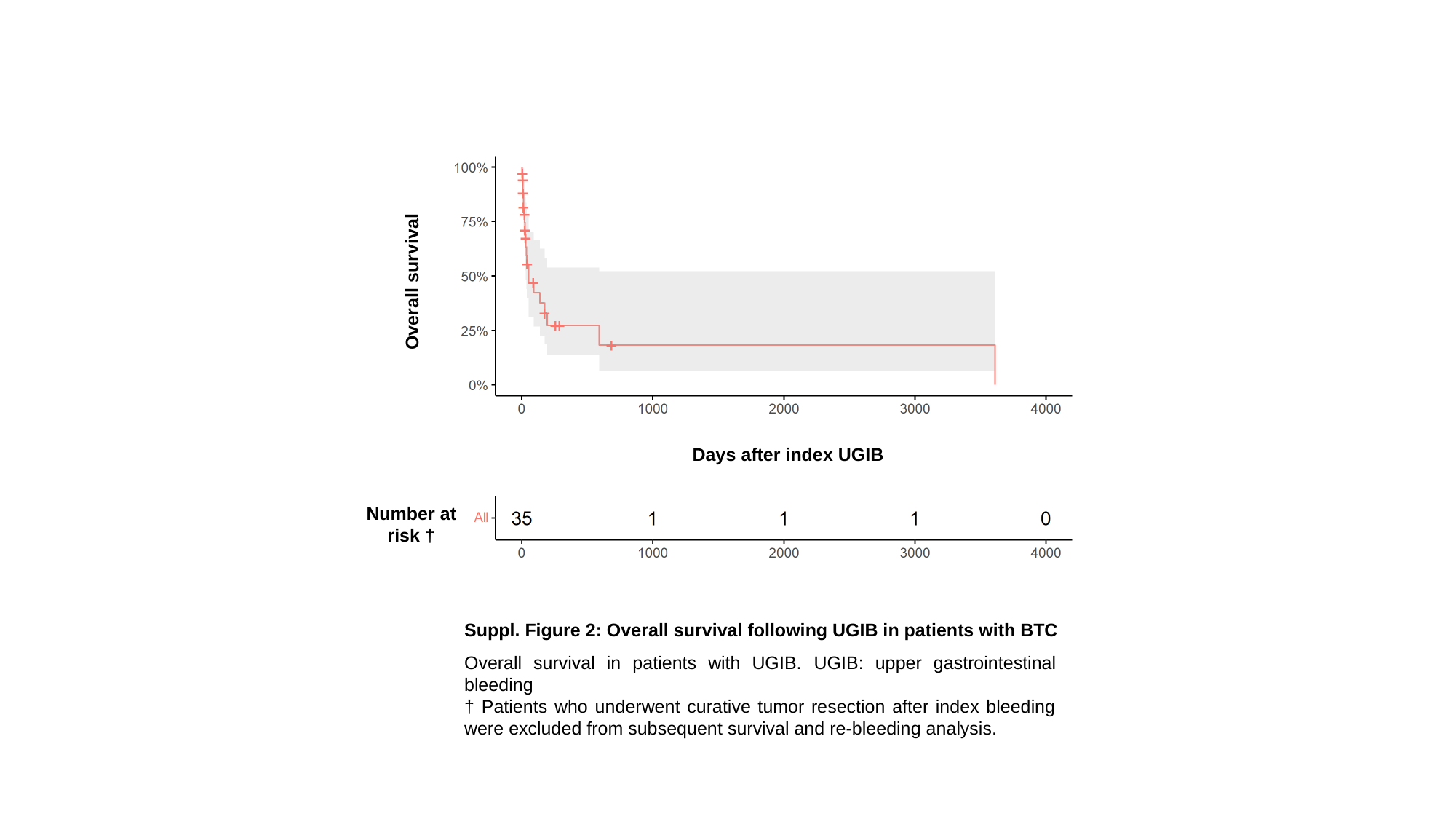

Overall survival
Days after index UGIB
Number at risk †
Suppl. Figure 2: Overall survival following UGIB in patients with BTC
Overall survival in patients with UGIB. UGIB: upper gastrointestinal bleeding
† Patients who underwent curative tumor resection after index bleeding were excluded from subsequent survival and re-bleeding analysis.
